# Automatic Target Recognition Strategy for Synthetic Aperture Radar Images Based on Combined Discrimination Trees

**DOI:** 10.1155/2017/7186120

**Published:** 2017-11-29

**Authors:** Xiaohui Zhao, Yicheng Jiang, Tania Stathaki

**Affiliations:** ^1^Research Institute of Electronic Engineering Technology, Harbin Institute of Technology, Harbin, Heilongjiang, China; ^2^Department of Electrical and Electronic Engineering, Imperial College, London, UK

## Abstract

A strategy is introduced for achieving high accuracy in synthetic aperture radar (SAR) automatic target recognition (ATR) tasks. Initially, a novel pose rectification process and an image normalization process are sequentially introduced to produce images with less variations prior to the feature processing stage. Then, feature sets that have a wealth of texture and edge information are extracted with the utilization of wavelet coefficients, where more effective and compact feature sets are acquired by reducing the redundancy and dimensionality of the extracted feature set. Finally, a group of discrimination trees are learned and combined into a final classifier in the framework of Real-AdaBoost. The proposed method is evaluated with the public release database for moving and stationary target acquisition and recognition (MSTAR). Several comparative studies are conducted to evaluate the effectiveness of the proposed algorithm. Experimental results show the distinctive superiority of the proposed method under both standard operating conditions (SOCs) and extended operating conditions (EOCs). Moreover, our additional tests suggest that good recognition accuracy can be achieved even with limited number of training images as long as these are captured with appropriately incremental sample step in target poses.

## 1. Introduction

Synthetic aperture radar (SAR) is a valuable technique for remote sensing and monitoring applications. Automatic target recognition (ATR) of SAR images is one of the most challenging SAR applications [1]. A typical SAR ATR system recognizes tactical ground targets of interests, that is, tanks, howitzers, and armoured vehicles, which is essential for identifying friends and foes and prerequisite for precision strikes.

SAR ATR involves a sequence of processes, such as some type of preprocessing, feature extraction, classifier construction, and finally target classification. The preprocessing stage may involve multiple types of processing that aims at facilitating the efficiency of image interpretation and analysis in the subsequent stages, for example, by suppressing the clutter reflections that obscure the contrast between the target of interest and the clutter. Moreover, SAR images are resized, shifted, and rotated to predefined standards. The so-called resizing is normally implemented by cropping out part of the image. The shifting and rotating processes are also known as image registration and pose rectification, respectively [2, 3].

Feature extraction is another essential stage which extracts effective discriminant features for improving recognition accuracy. Several features have already been exploited in SAR ATR [4–10]. Based on the consideration that tactical ground targets usually have a rectangular shape with different widths and lengths, geometric features are commonly used in SAR ATR. Zernike moments (ZMs) are employed in [6], taking advantage of their linear transformation invariance properties and robustness to the presence of noise. In [7], features are extracted based on pseudo-Zernike moments (pZm), which have merits such as the invariance properties, the independent property, and much lower sensitivity to noise in comparison with the ZMs. In [11], multiple geometric features are produced from calculating the axis projection of a target shape blob rotated clockwise with certain increment about the centre of the target. Then, the redundancy of the learned feature set is eliminated by keeping the rank of the covariance matrix of the new feature set the same as that of the entire data set. However, the geometric features of the target of interest in SAR images are difficult to measure precisely due to the cluttered background and variations in poses and depression angles. Therefore, the recognition accuracy is not guaranteed. The polar mapping method, which is frequently used in ISAR image classification, is modified and used in [3] to address the SAR ATR problem. The original images are converted from the original 2D spatial domain (range and cross-range) to images in the polar coordinate domain (radius and angle) to produce polar-mapped images. The polar-mapped images are similar to the images that are mapped from the same target even in different poses. For that reason, the commonly used pose estimator is not necessarily needed for polar-mapped images. However, the performance of the polar mapping method depends highly on the determination of the reference central point for coordinate transformation, which is not a simple task especially for SAR images captured under various clutter environments.

Certain features are not feasible to be directly applied to classification due to their high dimensionality [12–19]. In [12], a compact representation feature, the monogenic signal, is employed for SAR ATR, where the high dimensional problem is circumvented by uniform downsampling, normalization, and concatenation of the monogenic components. Feature dimensionality reduction methods for SAR ATR based on manifold learning theory are also studied in recent years [13–17]. In [16], each sample is given a weight, which is called the sample discriminant coefficient (SDC), relating to its similarity to neighbouring samples, and then the SDC is combined with the Local Discriminant Embedding (LDE) method for producing redundancy-reduced features. Similarly, in [17], the so-called neighbourhood geometric centre scaling embedding (NGCSE) method is proposed, where geometric centre scaling is introduced into the neighbourhoods such that the samples are provided with clear clustering directions. However, the performance of most of the nonlinear dimensionality reduction methods relies heavily on the parameter selection of the neighbourhood, which is still an open problem.

The nearest neighbour classifier is one of the most used classifiers, where the extracted features are directly fed into the classifier to achieve the classification results [16]. Sparse representation based classification (SRC) is recently developed and exploited in SAR ATR, where the feature vectors of the testing samples are coded as sparse linear combinations of the feature vectors of the training samples, and the target with the minimum residual energy is recognized [12, 20]. Methods such as Support Vector Machines (SVM), Neural Networks (NN), and adaptive boosting (AdaBoost) are all vastly exploited in SAR ATR [2, 5, 21–23]. Various choices of base learners can be combined with the AdaBoost algorithm to solve the SAR ATR problem [2]. As explained in the Hughes phenomenon (also known as the curse of dimensionality), the difficulty of constructing classifier models becomes more prominent especially when the feature set is high in dimensionality while the number of the training data is limited (a fact in SAR ATR). However, the combination of the AdaBoost and graphical models is empirically proven in [24] to demonstrate good performance even when the training data is limited in number.

**Figure 1:**
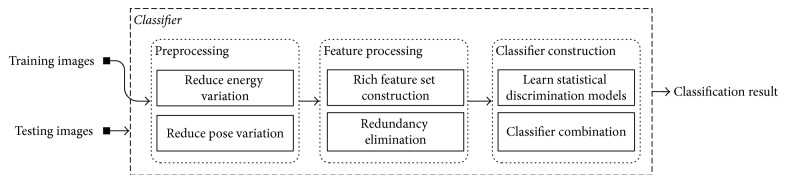
The SAR ATR scheme.

The SAR images are known for their indistinct appearances, variations in target appearances, and small number of available training samples. These problems must be properly addressed to achieve good recognition results for ATR tasks. To this end, a SAR ATR scheme is introduced as illustrated in Figure 1. Firstly, an initial processing stage is applied to facilitate the efficiency of feature extraction in the subsequent stages. More specifically, aiming at reducing the impact of variations in SAR images caused by variational echo energy and target poses, an image energy normalization process and a pose rectification process are applied sequentially. The construction of effective feature sets for ATR tasks is of crucial importance for achieving reliable recognition results. Therefore, it is suggested to extract a rich feature set that is formed by combining various types of discrimination features and then construct a more compact feature set by eliminating the redundancy of the rich feature set. We have decided to employ wavelet-based features. A rich feature set is firstly formed by combining the decomposed wavelet subband features, for example, the low-frequency information in LL subband coefficients and the high-frequency information in both LH and HL subband coefficients, where the HH subband is not involved since it is not stable feature in SAR images [25]. The involved coefficients actually depict the combination of texture features and horizontal and vertical edge features. After this, a compact low dimensional feature set which comprises features which retain most of the variance is constructed by employing the Principle Component Analysis (PCA) technique [26]. The relationship among features is statistically learned in a discriminative fashion rather than a generative fashion. Specifically, instead of using the true distribution, which is usually unknown for most of the time, the empirical estimates are learned in a discriminative fashion by maximizing the *J*-divergence. Therefore, although the learned models may have low consistency with the real model of target classes due to limited amount of training data, high discrimination ability can still be achieved. Then, a final classifier is constructed by combining several discriminative tree based classifiers with the Real-AdaBoost framework [27]. To evaluate the performance of the proposed method, the moving and stationary target acquisition and recognition (MSTAR) public release data set is involved. Experimental results demonstrate that the proposed method outperforms several widely cited methods under both standard operating conditions (SOCs) and extended operating conditions (EOCs).

Variation reduction techniques that facilitate the efficiency of feature extraction are introduced in Section 2. The feature extraction and processing techniques are introduced in Section 3. The recognition scheme is detailed in Section 4. Experimental results using the MSTAR public database are shown in Section 5, followed by our conclusions in Section 6.

## 2. Variation Reduction Techniques

### 2.1. Image Energy Normalization

The echo strength of SAR is strongly affected by, for example, the range distance between the imaging target and its corresponding radar and several other reasons; therefore the average amplitude of image pixels in different image chips may be different even for the same target [28]. To mitigate the potential influence of amplitude variations in subsequent features extraction, the image energy normalization process needs to be applied. Let *M* and *N* denote the number of pixels in range and cross-range dimension for a given SAR image chip. The SAR image chip can be denoted as *X*(*m*, *n*), where *m* = 1,…, *M* and *n* = 1,…, *N* are the dimension of range and cross-range, respectively. The energy normalized image pixel *X*′′(*m*, *n*) can be described as

(1) $$\begin{array}{c} X^{\prime \prime }\left(\;m,n\;\right)=\frac{X^{\prime }\left(m,n\right)-X_{min}^{\prime }\left(m,n\right)}{X_{max}^{\prime }\left(m,n\right)-X_{min}^{\prime }\left(m,n\right)}, \end{array}$$

where *X*_min_′(*m*, *n*) and *X*_max_′(*m*, *n*) is the minimum and maximum value among all pixels of *X*′(*m*, *n*), respectively, and *X*′(*m*, *n*) is calculated as

(2) $$\begin{array}{c} X^{\prime }\left(\;m,n\;\right)=\frac{X\left(m,n\right)}{\sqrt{\sum_{m=1}^{M}\sum_{n=1}^{N}X^{2}\left(m,n\right)}}. \end{array}$$

The benefit of employing the image energy normalization process is provided in Section 5.1.

### 2.2. Pose Rectification

Pose rectification is beneficial for improving the accuracy of SAR ATR and can be achieved by rotating the given images according to the pose of target of interests. However, targets with partial defected contour shapes that are caused by the shadow effect may suffer from poor pose estimation accuracies. This section introduces a pose estimation method that is based on the exploration of targets' geometrical information for achieving higher estimation accuracy.

Several methods have been proposed for achieving higher accuracy in pose estimation. The methods proposed in [29, 30] are based on maximizing the mutual information with multilayer perceptron (MLP). Although a low estimation error is achieved, these methods are computationally expensive and require a long training time. The method proposed in [31] is based on the 2D continuous wavelet transform (CWT), where the orientation that maximizes the angular energy is considered as the estimated pose. However, this method is based on the assumption that the target of interest is already placed in the image centre, which is difficult to achieve especially for SAR images with indistinct targets.

In fact, the tactical ground targets show rectangular shaped boundaries, which can be used for pose estimation. Therefore, methods based on the analysis of the geometrical information of target of interests have been proposed. The methods proposed in [2, 32] are based on finding the encapsulating box of the target of interest, where the basic assumption is that the edges of the estimated box should be tangent to the rectangular shaped target boundaries. However, this is not always true with incomplete target shape boundaries due to the shadow effects in SAR images. Moreover, the least squares linear fit based methods estimate the centreline of the target of interest, where the slope of the centreline is considered as the target pose. However, for similar reasons, the shadow effect in SAR may produce images with defected target, which can affect the corresponding pose estimation results. As discussed, the encapsulating box based methods have failed to achieve the optimum estimation result due to the defect targets in SAR images. However, as will be introduced, the Radon transform based method can achieve better estimation result in such scenarios [33]. Therefore, better estimation accuracy can be achieved by employing these two methods in a well-designed fashion. Firstly, the target of interest is segmented from the SAR image, and the rectangle that has the minimum perimeter around the segmented target is considered as the minimum bounding rectangle (MBR) [34]. Then, the completeness of the target of interest can be evaluated. In the case of a target with complete contour shape in the SAR image, the MBR estimated result is considered as the final result. Otherwise, the Radon transform is conducted and its estimation is used as the final result.

#### 2.2.1. Estimation for Targets with Complete Contour Shapes

**Figure 2:**
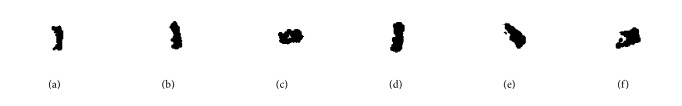
Illustration of targets with complete contour shapes.

Tactical targets in SAR images have randomly distributed poses ranging from 0° to 360° (the target pose is defined as the angle between the target's longer edge and the horizontal image axis). Tactical targets in SAR images show rectangular-like shapes.  Figure 2 shows the segmented SAR chips, where the target poses can be estimated according to the inclination angle of its MBR. As introduced in [34], the rectangle that has the shortest perimeter enclosing a convex polygon has at least one side collinear with one of the convex edges. The MBR can be efficiently calculated as follows:

 
*Step 1*. Estimate the centroid of the target of interest.
 
*Step 2*. Compute the convex polygon of the target of interest.
 
*Step 3*. Compute and store the edge orientations of the convex polygon.
 
*Step 4*. Rotate a bounding rectangle according to the stored edge orientations until a full rotation is done.
 
*Step 4.1*. Find a fitted rectangle.
 
*Step 4.2*. Store the perimeter of the fitted rectangle.
 
*Step 4.3*. Rotate the rectangle.
 
*Step 5*. Return the rectangle corresponding to the minimum perimeter.

#### 2.2.2. Estimation for Targets with Incomplete Contour Shapes

**Figure 3:**
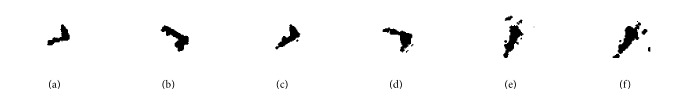
Illustration of targets with defected contour shapes.

Due to the imaging principle of SAR, partial part of the target of interest is not radiated by radar beam, and therefore the imaged target shows incomplete boundary shape. However, the long edge of the target of interest is always well imaged, as shown in Figure 3. In fact, the Radon transform (RT) can be used for long edge detection. Therefore, for SAR images with targets that show incomplete contour shapes, the RT based estimation can achieve higher accuracy. The application of the RT on a target image **I**(*x*, *y*) limited by a set of angles can be considered as calculating the projection of the target along given angles. The calculated projection result is the sum of pixel numbers in each single direction, where a line can be found in the corresponding target image according to the peak of the projection result [33]. Define **G**(*ρ*, *θ*) as the projection at angle *θ* with distant *ρ* to the image centroid, and the RT is implemented as follows:

(3) Gρ,θ=∫−∞∞∫−∞∞Ix,yδρ−xcos⁡θ−ysin⁡θdx dy,

where *δ*(·) is the Dirac delta function. The parameters *ρ* and *θ* determine the projection direction, where the projection is repeated from *θ* ∈ [0° : 180°). Note that a pixel in the RT transform is divided into four subpixels such that accurate projection result can be achieved, where the projection contribution is calculated according to the position of the subpixel that hits the projection bin.

#### 2.2.3. Degree of Overlapping Rectangle

**Figure 4:**
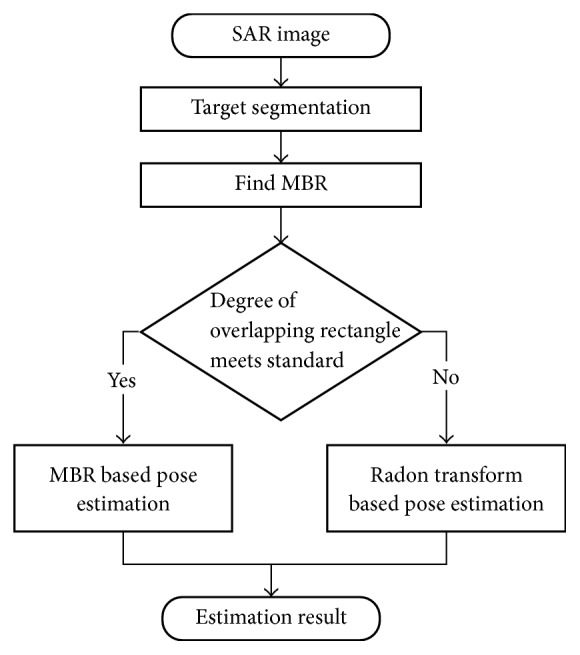
Illustration of the proposed pose estimation method.

**Figure 5:**
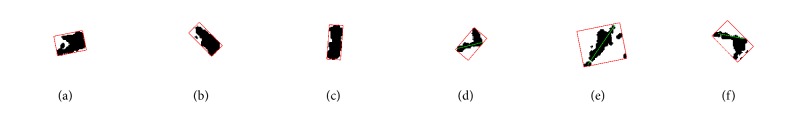
Illustration of the proposed pose estimation method, where the red rectangle is the MBR and the green line is the estimation result of the Radon transform. Note that the Radon transform is not used when the degree of overlapping rectangle meets the specified standard.

In fact, for any given image, the completeness of the target in SAR images can be automatically calculated. As introduced in Section 2.2.1, the calculated MBR has at least one edge overlap with the target boundary. Therefore, in the case of a complete target, one long edge of the target of interest will overlap with that of its corresponding MBR. In the case of a target with partial defect, the diagonal line of the target of interest may overlap with a long edge of its corresponding MBR with few pixels. Let *N*_*l*_ denote number of pixels of the two MBR long edges, and let *N*_*t*_ denote the number of target pixels that overlap with the two MBR long edges. The completeness of the target in SAR images can be evaluated as follows: the target is firstly dilated, and then the degree of overlapping rectangle is calculated as *N*_*t*_/*N*_*l*_, and finally the completeness of the target boundary is evaluated according to the calculated degree of overlapping rectangle. After dilation, since the difference between the complete contour shape and defected contour shape is large, the proposed method is not sensitive to the selected threshold employed for evaluating the degree of overlapping. Overall, as shown in Figure 4, the target pose is estimated using the MBR based method or the RT based method depending on the evaluation result of the degree of overlapping rectangle, and several estimation results are shown in Figure 5.

## 3. Feature Extraction and Processing Techniques

### 3.1. Rich Feature Set Extraction

**Figure 6:**
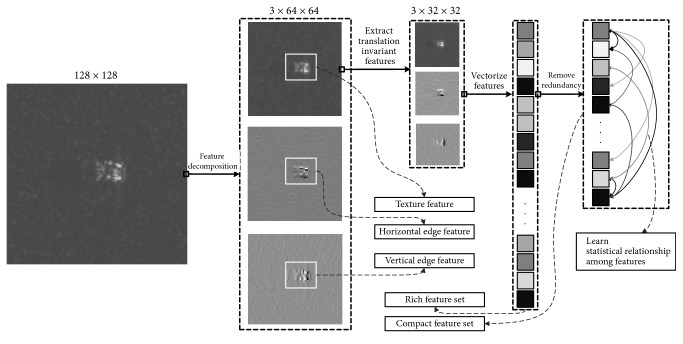
Illustration of the proposed feature extraction and processing technique.

**Figure 7:**
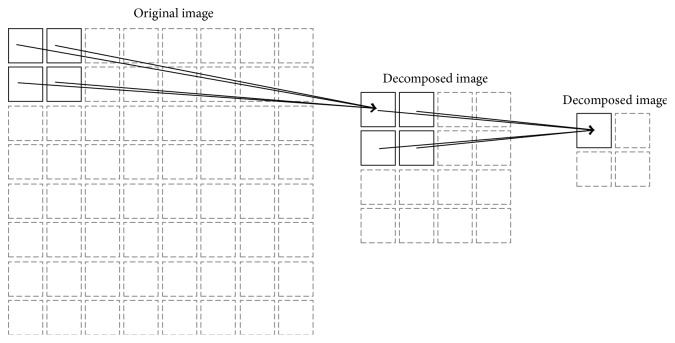
A single pixel point in the decomposed image depicts the existence of features in a corresponding entire local region of the original image.

Feature extraction is of crucial importance to the overall performance of the entire ATR system. It is ideally preferable to extract features that have characteristics of high discrimination ability (or, in other words, high interclass variation) and high tolerance to target translation. These feature characteristics can be achieved by efficiently employing the wavelet decomposition technique. As depicted in Figure 6, the texture features are reflected in LL and the horizontal and vertical edge feature are reflected in LH and HL, respectively. HH is actually a combination of features reflected in LH and HL. Furthermore, the translation invariant features can be extracted by sequentially further decomposing the previously decomposed image to a much coarser resolution. The idea behind the translation invariant features is that each decomposition process throws away the exact positional information of certain feature that exists in a specific area. More specifically, as illustrated in Figure 7, a pixel point in a newly decomposed image implicitly reflects the presence of certain feature(s) in a corresponding entire local region in the original image.

Several wavelet families have been proposed with the shape and duration of the mother wavelets being the main differences among them. The number of vanishing moments (order number) is used as an indication of the wavelets' smoothness and the frequency response flatness of the wavelet filters. It is suggested that we employ one fixed mother wavelet for the entire recognition scheme. A wavelets' comparison test is conducted in [5], where 7 mother wavelets with variations in order numbers are compared, according to minimum distance. To determine the most appropriate mother wavelet, in this paper, we compare 7 mother wavelets with more variations in order numbers with the maximum margin criterion (MMC) [35]. Specifically, we compare discrete Meyer wavelet, Biorthogonal wavelets (orders 1.1, 1.3, 1.5, 2.2, 2.4, 2.6, 2.8, 3.1, 3.3, 3.5, 3.7, 3.9, 4.4, 5.5, and 6.8), Coiflets (orders 1, 2, 3, 4, and 5), Haar wavelet, Daubechies wavelets (orders 2, 3, 4, 7, 10, 25, and 45), Reverse biorthogonal wavelets (orders 1.1, 1.3, 1.5, 2.2, 2.4, 2.6, 2.8, 3.1, 3.5, 3.7, 3.9, 4.4, 5.5, and 6.8), and Symlets (orders 2, 4, 8, and 16).

MMC finds the mother wavelet that maximizes the average margin between classes. This is achieved by comparing the difference between the average within-class distance and the average between-class distance *d*_*w*_ − *d*_*b*_. The mother wavelet that achieves the maximum difference is the best selection. Suppose we have *c* classes *C*_1_, *C*_2_,…, *C*_*c*_, each class with *n*_*i*_ samples and therefore, *n* = ∑_*i*=1_^*c*^*n*_*i*_ samples in total. Let *x*_*j*_^*i*^ denote the *j*th sample in the *i*th class, let *m*_*i*_ be the centroid of the *i*th class, and let *m* be the centroid of the training set. The average within-class distance *d*_*w*_ and the average between-class distance *d*_*b*_ can be denoted as

(4) dw=1n∑i=1c ∑j=1nixji−miF2db=1n∑i=1cnimi−mF2.

**Figure 8:**
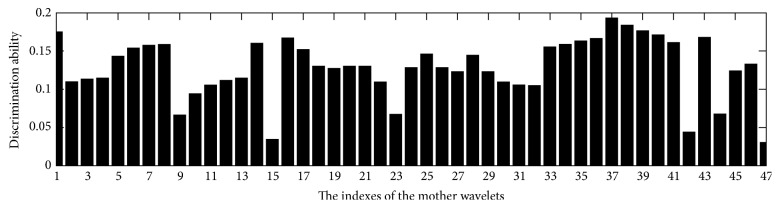
Discrimination ability of various types of mother wavelets. The involved 47 mother wavelets are sequentially discrete Meyer wavelet, Biorthogonal wavelets (orders 1.1, 1.3, 1.5, 2.2, 2.4, 2.6, 2.8, 3.1, 3.3, 3.5, 3.7, 3.9, 4.4, 5.5, and 6.8), Coiflets (orders 1, 2, 3, 4, and 5), Haar wavelet, Daubechies wavelets (orders 2, 3, 4, 7, 10, 25, and 45), Reverse biorthogonal wavelets (orders 1.1, 1.3, 1.5, 2.2, 2.4, 2.6, 2.8, 3.1, 3.5, 3.7, 3.9, 4.4, 5.5, and 6.8), and Symlets (orders 2, 4, 8, and 16). The 37th mother wavelet is the Reverse biorthogonal wavelet 3.1.

The comparison of the discrimination performance of the mentioned wavelets is illustrated in Figure 8. It is noted that the Reverse biorthogonal wavelet 3.1 achieves the highest value, an observation which indicates that it has the highest discrimination ability among these wavelets. Therefore, the Reverse biorthogonal wavelet 3.1 is selected as the default mother wavelet for feature extraction in SAR images.

The above process yields large sets of features which exhibit a high variability as far as the quality of the discriminative information that they convey is concerned. To achieve the truly effective features, the PCA is used, which achieves comparable result to both 2D-PCA and two-stage 2D-PCA when they are employed for SAR feature compression purposes, as analysed in [26]. Moreover, the PCA is much more efficient as far as both computation time and storage space are concerned. The implementation of the PCA is introduced as follows:

 
*Given*. Data *X* = [*x*_1_,…, *x*_*L*_]. Number of principal components *k*.
 
*Step 1*. Subtract the mean of variables from *X*.
 
*Step 2*. Solve the Singular Value Decomposition (SVD) of *X* = USV^*T*^.
 
*Step 3*. The dimensionality reduced feature set is calculated with the first *k* column of *V* as *XV*_*k*_.

### 3.2. Learn Statistical Relationship among Features

Since access to data arising from true distributions is often not available, the learned models based separately on positive/negative samples are usually not accurate enough for classification. In fact, the discriminative methods construct models from both the positively and negatively labelled samples in a discriminative fashion. Since the final objective is classification, even if the learned distributions may not converge to the true distributions, the constructed discriminative models tend to have better discrimination performance than the generative models [36, 37].

In binary classification case, which can be naturally extended to the more general *M*-ary classification case, for a given labelled training set *𝒮*≔{(*x*^(1)^, *y*^(1)^),…, (*x*^(*L*)^, *y*^(*L*)^)}, where *y*^(*l*)^ represents the sample label, each pair (*x*^(*l*)^, *y*^(*l*)^) ∈ *𝒳*^*n*^ × {+1, −1} (*𝒳* is normally a finite set of integer values as *𝒳* = {0,…, 255}). Supposing we have two models *p*(*x*)≔*P*_*X*∣*Y*_(*x*∣*y* = 1) and *q*(*x*): = *P*_*X*∣*Y*_(*x*∣*y* = −1) that can describe the true distribution of *p* and *q*, the log-likelihood ratio test is known to be the optimal test (under both the Neyman-Pearson and Bayesian settings [38])

(5) $$\begin{array}{c} \log\frac{p\left(x\right)}{q\left(x\right)}\overset{\hat{y}=+1}{\underset{\hat{y}=-1}{≷}}\eta , \end{array}$$

where *η* is the threshold [38].

In most cases, it is impossible to have access to the true conditional distributions *p* and *q*. Approximations $\hat{p}$ and $\hat{q}$ are normally built to learn the unknown distribution from the labelled training set *𝒮*. Therefore, the log-likelihood ratio test can be rewritten as

(6) $$\begin{array}{c} \log\frac{\hat{p}\left(x\right)}{\hat{q}\left(x\right)}\overset{\hat{y}=+1}{\underset{\hat{y}=-1}{≷}}\eta . \end{array}$$

The recently proposed method named discriminative tree estimates the multivariate distributions $\hat{p}$ and $\hat{q}$ jointly from both the positively and negatively labelled samples in the training set *S* of Tan et al. [37]. This method is based on the assumption that the learned distribution $\hat{p}(x)$ is Markov with respect to an undirected graph *𝒢* = (*𝒱*, *ℰ*), where *𝒱* = {1,…, *n*} represents the vertex set and $ℰ\subset \left(\begin{array}{c} 𝒱\\ 2 \end{array}\right)$ represents the set of all unordered pairs of vertexes. The mentioned Markov conforms to the local Markov property

(7) $$\begin{array}{c} p\left(\;x_{i},x_{V\backslash i}\;\right)=p\left(\;x_{i},x_{N\left(i\right)}\;\right),\;\forall i\in V, \end{array}$$

where *𝒩*(*i*)≔{*j* ∈ *𝒱* : (*i*, *j*) ∈ *ℰ*} represents the set of neighbour nodes of *i* and *x*_*𝒜*_ = {*x*_*i*_ : *i* ∈ *𝒜*} for any set *𝒜* ⊂ *𝒱*.

A tree structured distribution $\hat{p}$ that is Markov with respect to an undirected graph *𝒢* = (*𝒱*, *ℰ*) can be factorized as follows [39]:

(8) $$\begin{array}{c} \hat{p}\left(\;x\;\right)=\underset{i\in V}{\prod }{\hat{p}}_{i}\left(\;x_{i}\;\right)\underset{\left(i,j\right)\in E}{\prod }\frac{{\hat{p}}_{i,j}\left(x_{i},x_{j}\right)}{{\hat{p}}_{i}\left(x_{i}\right){\hat{p}}_{j}\left(x_{j}\right)}, \end{array}$$

where ${\hat{p}}_{i}(x_{i})$ represents the marginal of the random variable *x*_*i*_ and ${\hat{p}}_{i,j}(x_{i},x_{j})$ represents the pairwise marginal of the pair (*x*_*i*_, *x*_*j*_).

Based on this, for a given distribution *p*, the projection of *p* onto some tree distribution *𝒢* = (*𝒱*, *ℰ*) is defined as follows:

(9) $$\begin{array}{c} \hat{p}\left(\;x\;\right)≔\underset{i\in V}{\prod }p_{i}\left(\;x_{i}\;\right)\underset{\left(i,j\right)\in E}{\prod }\frac{p_{i,j}\left(x_{i},x_{j}\right)}{p_{i}\left(x_{i}\right)p_{j}\left(x_{j}\right)}. \end{array}$$

We digress here to introduce the method for constructing models in generative fashion and then provide the method for constructing models in discriminative fashion. The generative methods attempt to construct a model that is the same as the underling model of the classification target. The widely researched generative method, namely, the Chow-Liu algorithm [40], employs the KL-divergence as the measure of the differences between two probability distributions *p* and $\hat{p}$. The optimization in the Chow-Liu algorithm is therefore defined as

(10) $$\begin{array}{c} \underset{\hat{p}\in T}{\min}\;D\left(\;p||\hat{p}\;\right)≔\underset{\hat{p}\in T}{\min}\;E_{p}\log\left(\;\frac{p}{\hat{p}}\;\right), \end{array}$$

where $\hat{p}\in 𝒯$ states that $\hat{p}$ is a tree structured distribution over the same alphabet as *𝒯*. It is shown by Chow and Liu that this optimization problem can be solved by using a maximum weight spanning tree (MWST) algorithm (e.g., Kruskal's [41]) where the mutual information is used to represent the edge weights between pairs of variables.

In contrast, the recently proposed discriminative method employs the *J*-divergence as the measure of the separation between two probability distributions *p* and *q*. The *J*-divergence is defined as follows [42]:

(11) $$\begin{array}{c} J\left(\;p,q\;\right)≔D\left(\;p||q\;\right)+D\left(\;q||p\;\right). \end{array}$$

The optimization problem reduces to two tractable MWST problems for maximizing the tree approximate *J*-divergence over the two tree structured-distributions $\hat{p}$ and $\hat{q}$ for known empirical distributions $\tilde{p}$ and $\tilde{q}$, which is defined as

(12) $$\begin{array}{c} \left(\;\hat{p},\hat{q}\;\right)=\underset{\hat{p}\in T_{\tilde{p}},\hat{q}\in T_{\tilde{q}}}{\mathrm{argmax}}\hat{J}\left(\;\hat{p},\hat{q};\tilde{p},\tilde{q}\;\right), \end{array}$$

where

(13) $$\begin{array}{c} \hat{J}\left(\;\hat{p},\hat{q};\tilde{p},\tilde{q}\;\right)≔\underset{x\in X^{n}}{\sum }\left(\;\tilde{p}\left(\;x\;\right)-\tilde{q}\left(\;x\;\right)\;\right)\log\left[\;\frac{\hat{p}\left(x\right)}{\hat{q}\left(x\right)}\;\right]. \end{array}$$

It is noted that, as described in [37], (13) can be decoupled into two independent optimization problems:

(14) p^=argminp∈Tp~⁡ Dp~||p−Dq~||pq^=argminq∈Tq~⁡ Dq~||q−Dp~||q.

These can be solved by the MWST algorithm

(15) $$\begin{array}{c} \psi_{i,j}^{\left(+\right)}≔E_{{\tilde{p}}_{i,j}}\left[\;\log\frac{{\tilde{p}}_{i,j}}{{\tilde{p}}_{i}{\tilde{p}}_{j}}\;\right]-E_{{\tilde{q}}_{i,j}}\left[\;\log\frac{{\tilde{p}}_{i,j}}{{\tilde{p}}_{i}{\tilde{p}}_{j}}\;\right]. \end{array}$$

Overall, the procedure of the learning of the discriminative tree is summarized in the following steps [37]:

 
*Given*. Training set *𝒮*.
 
*Step 1*. Estimate the pairwise statistics ${\tilde{p}}_{i,j}(x_{i},x_{j})$ and ${\tilde{q}}_{i,j}(x_{i},x_{j})$ for all edges (*i*, *j*).
 
*Step 2*. Calculate edge weights {*ψ*_*i*,*j*_^(+)^} and {*ψ*_*i*,*j*_^(−)^} for all edges (*i*, *j*).
 
*Step 3*. Find the optimal tree structures with the given edge weights.
 
*Step 4*. Set $\hat{p}$ and $\hat{q}$ to be the projection of $\tilde{p}$ onto ${ℰ}_{\hat{p}}$ and $\tilde{q}$ onto ${ℰ}_{\hat{p}}$, respectively.
 
*Step 5*. Classify the test sample *x* using the learned distributions $\hat{p}$ and $\hat{q}$ in a likelihood ratio test $h\left(x\right)=sgn[\log\left(\hat{p}(x)/\hat{q}(x)\right)]$.

Since the classification result is finally determined by the numerical result of the log-likelihood ratio test, we choose to employ one fixed threshold 0 for the entire training process. This is because likelihoods larger than 0 indicate higher probability of belonging to *p*(*x*). Similarly, likelihoods smaller than 0 indicate high probability of belonging to *q*(*x*).

## 4. Recognition Scheme

The main aim of classifier construction in ATR is to convert a wealth of training data into useful knowledge for classification by learning. However, a classifier learned from massive amounts of high varying data is not guaranteed to achieve good performance in classification and may yield large feature dimensions. Therefore, it is of great importance to find effective representations for the targets of interest to be used for constructing the classifiers.

**Figure 9:**
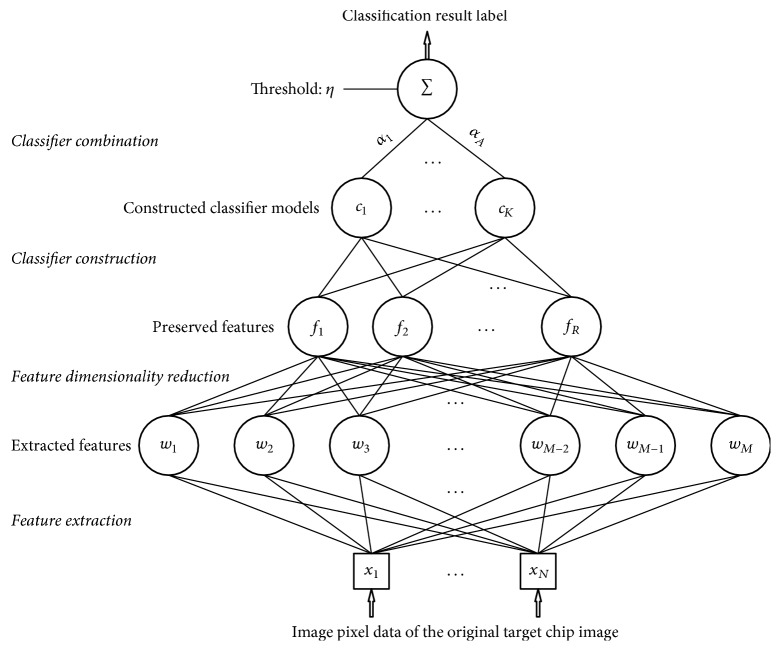
A generic diagram that depicts the various steps of the proposed SAR ATR scheme.

Extracted features might comprise large sets of features which at a glance might be worth of exploiting but turn out to be too “messy” and high in redundancy. In fact, the learning process of the classifiers could be enormously benefited from a feature dimensionality reduction process after the acquisition of the extracted features as previously discussed. The redundancy-reduced features can be used for learning classifiers, where efficient classifiers and better classification accuracy results can be achieved. Therefore, it is suggested to enlarge the quantity of the potential features but then eliminate the existing redundancy, reduce the dimensionality of the enlarged feature set, and finally exploit the preserved features for classification, which comprise the characteristics of proper combination of both quality and quantity. In the proposed recognition scheme, features are extracted with wavelet decomposition, but then the dimension of the feature set is reduced to provide a feature set rich in discriminative information but with limited dimensionality and less redundancy. To make the most of the extracted features, tree structured classifiers are learned in discriminative fashion based on the statistical information provided by the training data of the target classes. In the learned classifiers, the feature nodes are connected as a spanning tree, where each node is connected to another node which has the maximum relevance. Moreover, the relevance between feature nodes can be accordingly calculated. Finally, classifiers are combined using the Real-AdaBoost algorithm to construct the final classifier that has high classification accuracy and is less prone to overfitting, where the recently proposed discriminative trees are involved as the base classifiers. A generic sequence of steps of the proposed scheme is illustrated in Figure 9.

### 4.1. Construct a Strong Classifier

Efforts have been constantly made to construct a classifier with high classification accuracy and strong generalization ability (the later meaning that performance of the classifier learned from a given training dataset will still be good when the classifier is exposed to unseen data) [43]. Employing ensemble learning methods is one of the solutions. Ensemble learning methods construct and combine a set of base classifiers instead of constructing and using one single classifier learned from the training dataset. Base classifiers can be generated from a training dataset with the use of any learning algorithm (e.g., decision tree, graphical models, and neural networks).

AdaBoost is one of the ensemble methods that have achieved great success in diverse domains [27, 43–47]. The general idea of the AdaBoost is to constantly update the distribution of the training data such that the learning of the base classifiers in each iteration focuses more on the wrongly labelled samples by the previous learned base classifiers. Real-Adaboost is a variant of the AdaBoost which has been empirically proved to have better performance than ordinary AdaBoost (Discrete-AdaBoost) [27, 37, 44]. Specifically, for a given training dataset *𝒮*, each sample is assigned with an initial weight *ω*_0_^(*l*)^ = 1/*L*, where *L* is the number of training samples. A base classifier is learned in each iteration *t* such that *h*_*t*_ : *𝒳*^*n*^ → *ℝ*, where a larger absolute value in *h*_*t*_(*x*) indicates higher confidence. Then, the samples wrongly labelled by *h*_*t*_(*x*) are increased in weights such that the constructed classifiers in the following iterations can focus on the misclassified samples. Finally, the combined classifier resulting after *T* iterations is

(16) $$\begin{array}{c} H_{T}\left(\;x\;\right)=sgn\left[\;\overset{T}{\underset{t=1}{\sum }}\alpha_{t}h_{t}\left(\;x\;\right)\;\right], \end{array}$$

where sgn is the sign function that sgn(*a*) = 1 if *a* ≥ 0 and −1 otherwise and *α*_*t*_ is the coefficient calculated in each iteration for minimizing the weighted training error. Overall, the Real-AdaBoost algorithm trains a set of base classifiers sequentially and combines them to a strong classifier, where the current learned base classifiers focus more on the wrongly labelled samples by the previous base classifiers.

The ensemble process of the Real-AdaBoost is iterated with the rearrangement of the training set distribution while the learning method of the base classifiers is not changed. For the learning of the base classifier in each iteration *t*, the group of redundancy and dimensionality reduced wavelet features are employed and fed to learn the discriminative trees for classifier construction. By employing the learning method introduced in Section 3.2, a pair of discriminative trees is constructed to provide an estimation of the classification result. Specifically, the pair of discriminative trees constitutes a base classifier for the Real-AdaBoost *h*_*t*_ : *𝒳*^*n*^ → *ℝ*, where $h_{t}(x)=\log\left[{\hat{p}}_{t}(x)/{\hat{q}}_{t}(x)\right]$, and ${\hat{p}}_{t}$ and ${\hat{q}}_{t}$ denotes the learned discriminative tree models at the *t*th iteration of the Real-AdaBoost. After *T* iterations, *T* pairs of discriminative trees are learned and combined to construct a stronger classifier with better approximation of the classification result which can be written as Viola and Jones [45]

(17) HTx=sgn∑t=1Tαtlog⁡p^txq^tx=sgnlog⁡∏t=1Tp^txαt∏t=1Tq^txαt=sgnp^∗xq^∗x,

where ${\hat{q}}^{*}(x)=\prod_{t=1}^{T}{\hat{p}}_{t}(x{)}^{\alpha_{t}}$ and ${\hat{q}}^{*}(x)=\prod_{t=1}^{T}{\hat{q}}_{t}(x{)}^{\alpha_{t}}$.

For the iterative updating of the training set distribution, the misclassified samples are reassigned with larger weights and the correctly classified samples are reassigned with smaller weights compared to their previous weights. Regarding the weight distribution updating problem, simply reduplicating the samples with higher weights is time and computation inefficient. This is because as the number of iterations increases, the wrongly labelled samples would be much less in number but have much larger weights. Therefore, the final training set is fixed in size and constructed in random sampling fashion, where samples of the original training set are chosen according to the updated distribution weights. The entire classifier construction scheme is summarized as below:

*Given*. Training dataset *S*. Number of iterations *T*.
*Step 1*. Wavelet feature extraction from the given training dataset *S*.
*Step 2*. Redundancy and dimensionality reduction for the extracted features.
*Step 3*. Initialization of the distribution weights, *w*_0_^(*l*)^ = 1/*L* for all 1 ≤ *l* ≤ *L*.
*Step 4*. Classifier construction
 (1)* for t* = 1 : *T do*
  (2) Learn the pair of discriminative trees ${\hat{p}}_{t}$, ${\hat{q}}_{t}$ from the weighted empirical distributions ${\tilde{p}}_{w}$ and ${\tilde{q}}_{w}$.
  (3) Get the base classifier $h_{t}(x):=\log\;[{\hat{p}}_{t}(x)/{\hat{q}}_{t}(x)]$.
  (4) Calculate the coefficient *α*_*t*_
  (18) $$\begin{array}{c} \alpha_{t}=\frac{1}{2}\log\frac{1-\sum_{i=1}^{L}w_{t}^{\left(l\right)}y^{\left(l\right)}sgn\left(h_{t}\left(x^{\left(l\right)}\right)\right)}{\sum_{i=1}^{L}w_{t}^{\left(l\right)}y^{\left(l\right)}sgn\left(h_{t}\left(x^{\left(l\right)}\right)\right)}. \end{array}$$
  (5) Update the weighted empirical distribution:
  (19) $$\begin{array}{c} w_{t+1}^{\left(l\right)}\left(\;i\;\right)=\frac{w_{t}^{\left(l\right)}\exp\left[-\alpha_{t}y^{\left(l\right)}h_{t}\left(x^{\left(l\right)}\right)\right]}{\zeta_{t}},\;\forall l=1,\ldots ,L, \end{array}$$
where *ζ*_*t*_ is the normalization factor (to ensure that *w*_*t*+1_^(*l*)^ will be a distribution).
 (6)* end for*
*Step 5*. Output the final classifier $h_{t}(x)=\log\;[{\hat{p}}_{t}(x)/{\hat{q}}_{t}(x)]$ with coefficients {*α*_*t*_}_*t*=1_^*T*^.

### 4.2. Multiclass Classification

The One-vs.-One (OvO) and One-vs.-All (OvA) are the two most popular strategies for the extension of a two-class classification (binary classification) problem to a multiclass classification (multinomial classification) [48]. For a *K* class problem, the OvO strategy trains *K*(*K* − 1)/2 binary classifiers, each of which classifies a pair of classes selected from the original training set. For the classification of the unseen samples, the samples are fed and tested in all *K*(*K* − 1)/2 classifiers by employing a voting scheme where the class which achieves the highest number of positive predictions would be considered as the final prediction. The OvA strategy trains one classifier for every class where the samples of the target class are considered as positive samples and all of the rest of the samples as negative samples. At predication stage, the unseen sample is assigned with the label of class *k* if its corresponding classifier produces the highest likelihood score.

## 5. Experimental Results

In this section, the performance of the proposed scheme is evaluated and compared with several established methods. The widely used SAR ATR experimental validation and comparison benchmark moving and stationary target acquisition and recognition (MSTAR) public release database is employed for performance evaluation [49–51]. The MSTAR database consists of 10 vehicle classes, which are collected by X-band SAR with 1-ft by 1-ft resolution, including BMP2, BTR70, T72, BTR60, 2S1, BRDM2, D7, T62, ZILI131, ZSU234, and SLICY. The collection of target images is captured under various depression angles and aspect angles, which are suitable for testing the SAR ATR methods with targets under various operating conditions.

There are two categories of operating conditions in the MSTAR database: the standard operating conditions (SOCs) and the extended operating conditions (EOCs) [50]. The targets captured under SOCs are listed in Table 1 including information about vehicle types, number of chip images, serial numbers, and depression angles. It is worth noting that the EOCs are much more difficult for SAR ATR than the SOCs. In EOC-1, the depression angles are larger in variation where the training images are captured under 15° and the testing images are captured under 30°, as shown in Table 2. In EOC-2, the training and testing set have various versions of T72 with different serial numbers, as shown in Table 3.

We experiment with both two-level and three-level two-dimensional wavelet decomposition with respect to the Reverse biorthogonal wavelet (the selected mother wavelet as introduced in Section 3.1). In the following, wavelet 768 (256 × 3 = 768) and wavelet 192 (64 × 3 = 192) are used to denote the two-level and three-level wavelet decomposition, respectively. The stopping criterion of the Real-AdaBoost is set to 400 iterations. The segmentation of the target of interest is implemented with the MRF model based method, where the potential class number is 2, the expectation is 0.4, and the maximum iteration number is 50. The segmented target is dilated with a disk-shaped template with radius 3. The degree of overlapping rectangle is 0.5 indicating that an appropriate MBR must have more than 50% long edge overlapping pixels in terms of the target of interest. Moreover, the proposed method is implemented using Matlab R2013a and tested on a computer with 1.8 GHz CPU and 4 GB RAM. Regarding the computation complexity, for a classifier trained for classifying 10 targets in OvO fashion, the processing time for one single sample takes less than 0.02 s, including the processes of extraction and compressing of features and recognition of targets.

Before applying the proposed method to SAR ATR and comparing with other methods, it is necessary to test the proposed method in conjunction with several important processes, including image energy normalization, feature extraction, extension of two-class to multiclass classification, and pose rectification. These four tests are conducted in Sections 5.1to 5.4, and the comparisons of recognition accuracy performance with other methods are provided in Section 5.5.

### 5.1. Image Energy Normalization

The significance of image energy normalization in SAR ATR is tested in this section, where the performance of the proposed scheme is tested with or without image energy normalization processing. The dataset includes all 10 classes captured under SOCs as listed in Table 1. The wavelet 192 is used for feature extraction.

**Figure 10:**
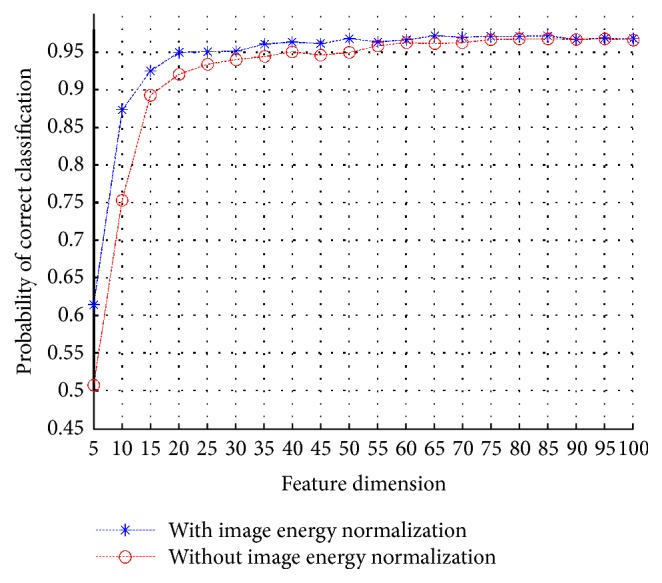
Performance comparison among the two cases which refer to the employment or not of image energy normalization.

It is noticed in Figure 10 that the involvement of normalization before feature extraction is beneficial for improving classification accuracy. In fact, as the dimension of feature vectors employed for classification grows, the advantage of image energy normalization diminishes. This is because a larger training feature set provides more information for classification, where the classifier is empowered with more discrimination ability by exploiting the provided information. However, the classification with normalization achieves good classification accuracy (around 96%) even when the feature vector dimension is much lower, yielding an accuracy which is almost the same as the accuracy achieved with higher feature dimensions. Therefore, it is still suggested to employ image energy normalization for preprocessing, especially for classifiers constructed from training feature sets of lower dimensionality. In the following, the image energy normalization process is employed as a standard default processing step.

### 5.2. Extension to Multiclass

**Figure 11:**
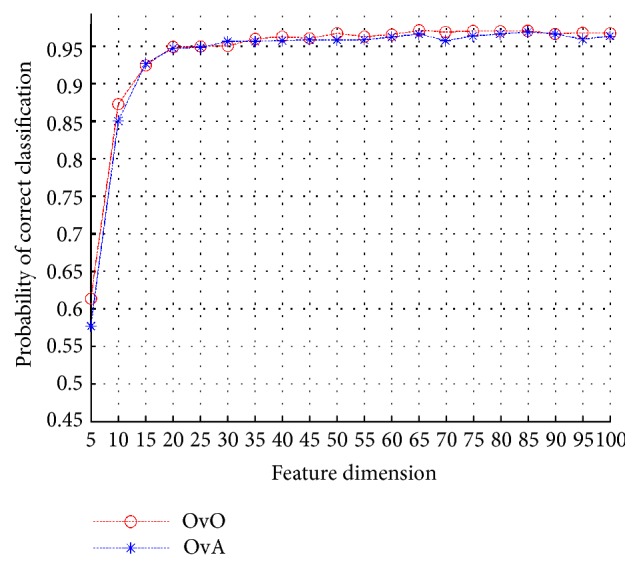
Performance comparison between OvO and OvA strategies.

We compare the OvO and OvA strategies on the same training set (all 10 classes under SOCs) to test their performance on the SAR ATR problem. It is noted in Figure 11 that the OvO strategy appears to be outperforming the OvA strategy marginally in the SAR ATR problem. The marginal differences in recognition accuracy lie in the unbalance of the training set, where the OvA strategy employs the positive sample classes that are much less in quantity than the negative sample classes. In fact, the advantage of the OvA strategy is that it is less in computation and time complexity, where the OvO constructs 45 classifiers and the OvA constructs 10 classifiers for a 10-class problem, respectively. Since the aim of this paper is to provide a SAR ATR scheme with high recognition accuracy, OvO is employed as the default strategy for solving the multiclass problem.

### 5.3. Feature Extraction

**Figure 12:**
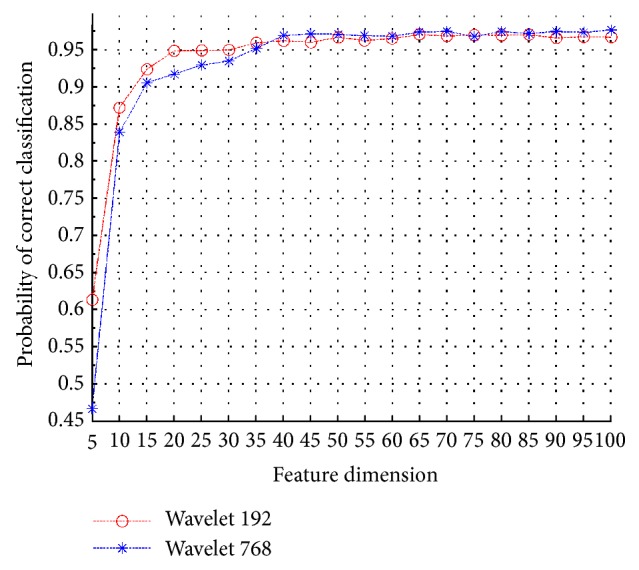
Performance comparison between wavelet 192 and wavelet 768.

In this section, we compare the performance of feature extraction using the wavelet 192 (three-level wavelet decomposition) and the wavelet 768 (two-level wavelet decomposition). All 10 classes captured under SOCs are employed for both training and testing. As illustrated in Figure 12, these two curves coupled with each other. The wavelet 192 outperforms the wavelet 768 when feature vectors possess lower dimensions. However, this situation changes as the dimension of feature vectors grows to 40. Moreover, the best classification result (97.46%) is achieved by the use of wavelet 768 with feature dimension of 70. This is because the wavelet 768 provides more features for discrimination and the proposed ATR scheme constructs and combines several discriminative tree classifiers that make the most of the discriminative information inherent to the extracted features.

### 5.4. Pose Rectification

#### 5.4.1. Pose Estimation

To test the performance of the proposed pose estimation method, the estimation results of the proposed methods are compared to the ground truth of target poses (the azimuth information provided in the MSTAR database). The correctness of the estimation results is evaluated with the so-called mean absolute difference (MAD), which is calculated as Err = 1/*n*∑_*i*=1_^*n*^|*E*_*t*_(*i*) − *E*_*e*_(*i*)|. The MAD reveals the actual estimation error about the ground truth in comparison to the mean error (ME), since it prevents the offset of the positive and negative errors. Moreover, the performance of the proposed method is evaluated and compared with several widely cited methods, such as the least square method (LSM) based estimation, the Hough transform (HT) based method, the MBR based method, and the Radon transform (RT) based method.

All 10 targets captured with different depression angles and target poses are involved to test the robustness of the proposed method over depression angle variations. The evaluation results for the 10 targets at depression angles 17° and 15° are listed in Tables 4 and 5, respectively. All serial number variants of BMP2 and T72 in MSTAR dataset are involved to test the robustness over variation in serial numbers. The evaluation results of the data captured at depression angles 17° and 15° are listed in Tables 6 and 7, respectively. It is noted that the MBR based method has achieved much lower estimation error in comparison to other methods. However, the performance of the MBR based method has estimation error higher than 10° in several tests. More specifically, the MBR based method achieves the highest estimation error 15.32° for the BRDM2 captured at depression angle of 15°. In comparison with these methods, the proposed method achieves the lowest estimation error in all tests (lower than 10°).

**Figure 13:**
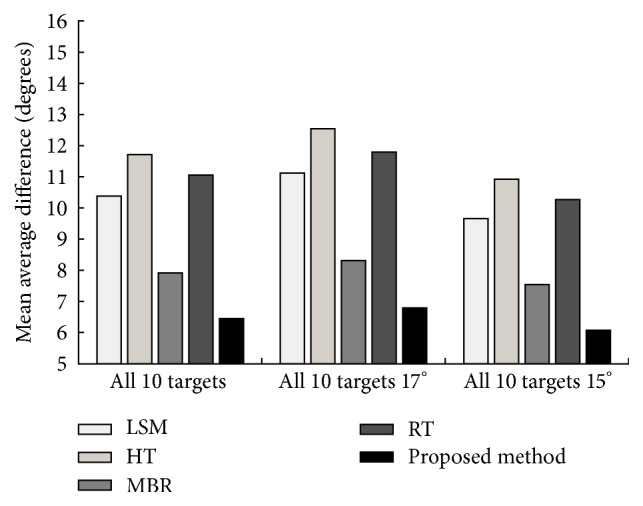
Performance comparison for all 10 targets at both depression angles of 17° and 15°.

**Figure 14:**
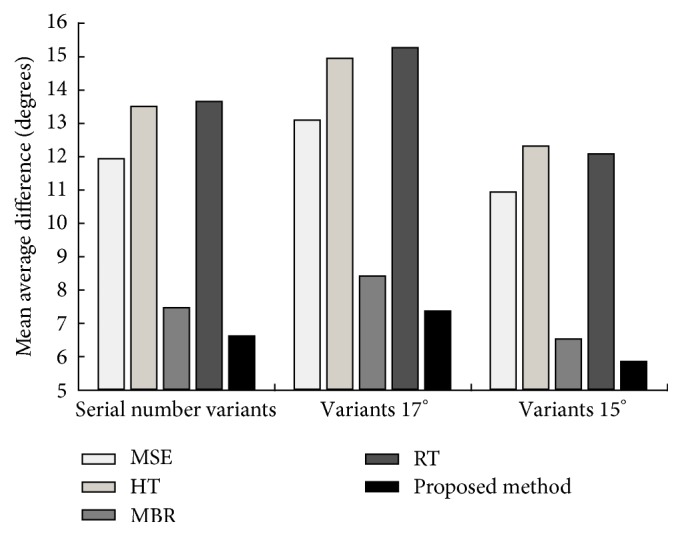
Performance comparison for serial number variants of BMP2 and T72 at both depression angles of 17° and 15°.

Furthermore, the average estimation error of the above tests is illustrated as bar figure in Figures 13 and 14, such that a much more distinct comparison can be observed. Similarly, the proposed method is compared to the least square method (LSM) based estimation, the HT based method, the MBR based method, and the RT based method. It is noted that, for most of these methods, higher estimation error is achieved in the serial number variation test. Compared with these methods, the proposed method achieves robust and accurate estimation results in both tests. Specifically, the proposed method achieves the lowest average estimation error in all tests (lower than 8°).

#### 5.4.2. Pose Rectification

**Figure 15:**
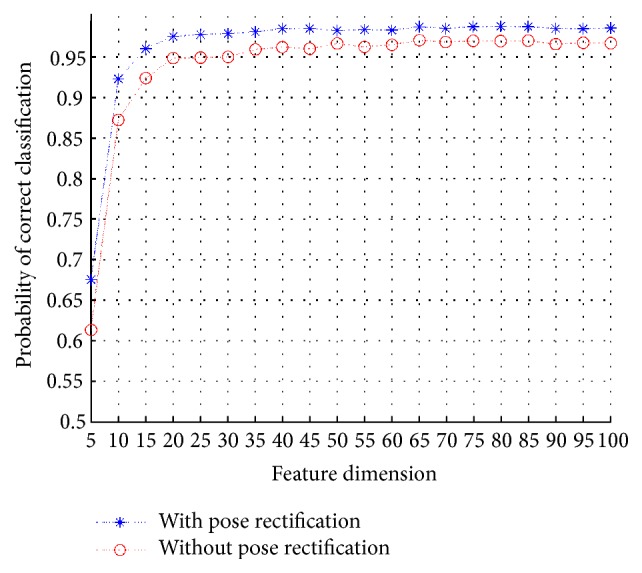
Performance comparison among the two cases which refer to the employment or not of pose rectification.

The various target poses introduce great variations into the SAR images. It has been experimentally proven in several researches that rotating images in certain directions or introducing rotationally invariant features is beneficial for improving classification accuracy [2, 3]. To this end, we rotate the image according to the target poses in the SAR images, which is named as pose rectification. In this section, we test the performance of the proposed scheme with or without pose rectification using the same training and testing set (all 10 classes under SOC). The SAR images are rotated anticlockwise according to their poses. As can be seen from Figure 15, the classification with pose rectification universally outperforms the classification without pose rectification. It is also noted that the best classification accuracy (99.3%) is achieved by the wavelet 768 with feature dimension of 75. These results meet our expectation that the classification can benefit from eliminating the pose variations in SAR images. Specifically, the rectification of poses provides target images for classification with fewer variations.

#### 5.4.3. Outlier Rejection Performance

**Figure 16:**
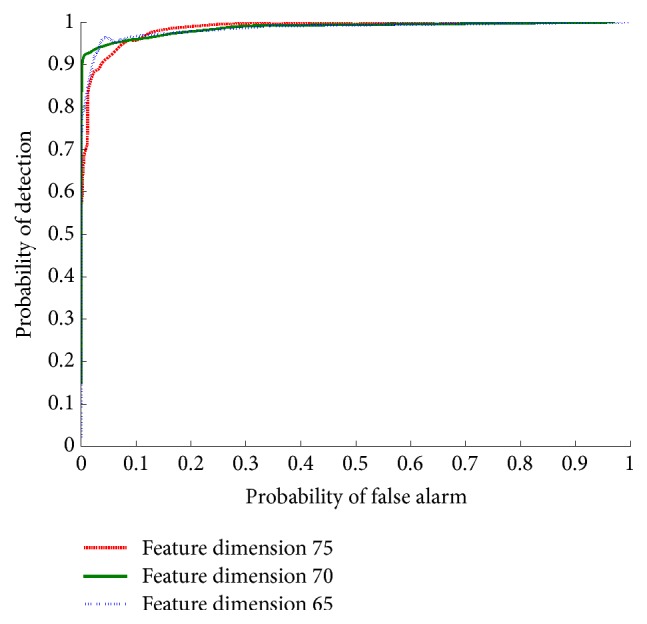
The outlier rejection performance of the proposed method with wavelet 768 compressed with different dimensions.

To evaluate the outlier rejection performance of the proposed method, a varying threshold for the log-likelihood test, which is introduced in Section 3.2, was incorporated to provide the ROC curve. BTR70, BMP2, and T72 listed in Table 1 are involved for classifier training and the SLICY set is involved as confusers with 1168 image chips, that is, 210 chips captured at 15°, 298 chips captured at 16°, 386 chips captured at 17°, and 274 chips captured at 29°. As shown in Figure 16, at the probability of detection *P*_*d*_ = 90%, the probability of false alarm for feature dimension of 75 is *P*_fa_ = 2.34%. It is clear that the proposed method is robust at rejecting confuser targets.

### 5.5. ATR Performance Comparisons

The effectiveness of the proposed ATR scheme is tested in this section. Several widely cited methods are involved for performance comparison, for example, the Extended Maximum Average Correlation Height Filter (EMACH) [53], the Support Vector Machine (SVM) classifier with Gaussian kernel [52], feature fusion via AdaBoost with neural networks as the base classifiers [2], and the Iterative Graph Thickening (IGT) approach [24]. The best result obtained from the proposed method is used to compare with other methods.

Table 8 lists the performance comparison of the mentioned methods under SOCs. It is noted that the proposed method has achieved significant improvements in classification accuracy in comparison with other methods. A majority of the classes are correctly classified with 100% accuracy and the rest have *P*_cc_ higher than 98%, which is also much higher than other methods. Moreover, the superiority of the proposed method is strengthened by the fact that the average *P*_cc_ (99.3%) is much higher than the second highest average *P*_cc_ (84.8%).

Four distinct target classes are involved in the following test: EOC-1, including 2S1, BRDM2, T72, and ZSU234, as listed in Table 2. All of these four classes are involved in training and testing stages. The only difference is that the training and testing set are captured under depression angles 15° and 30°, respectively. The increase in depression angle variations introduces a bigger challenge to the classification problem. It is noted in Table 9 that the classification accuracy of the most of the mentioned methods is lower than 88% under EOC-1, where the superiority of the proposed method (higher than 96%) is obvious. Furthermore, the average classification accuracy of the proposed method is 97.5% which is much higher than the other listed methods.

The training dataset under EOC-2 is composed of four different target classes, BMP2, BRDM2, BTR70, and T72, as summarized in Table 2. This test aims at testing the performance of the SAR ATR algorithms with significant different in serial numbers and configurations. The testing set has only the T72 family with five different serial numbers and the training set is composed of all these four mentioned classes. In addition, the training set was obtained at 17° while the testing set was obtained at depression angles of both 15° and 17° as shown in Table 3.  Table 10 lists the performance comparison of the mentioned methods under EOC-2. The improvement in classification accuracy is substantial since the average *P*_cc_ of the proposed method is 96.9% which is much higher than the second highest 84.8%.

### 5.6. Performance Comparison of Variations in Target Poses

**Figure 17:**
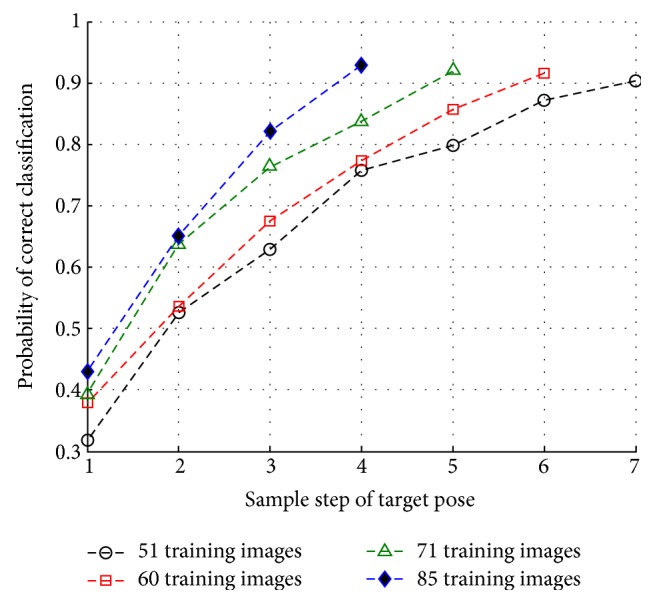
Performance comparison of training data selected with different incremental steps of target poses.

As analysed in Section 5.4, the involvement of pose rectification is beneficial for improving the classification performance. In fact, a single target will exhibit different appearances when it is captured under various poses. In this section, we conduct an experiment to test the influence of the appearance differences introduced by the pose variations. The experimental database is almost the same as the data listed in Table 1 except that only one single serial number of each target is involved for training and testing (C21 for BMP2 and S7 for T72). The images for training are selected from the training database with different sample steps of target poses (varied from 1° to 7°), where 51 images are selected for the training of each target. For example, the poses for Step 1 are 1°, 2°,…, 51°, the poses for Step 2 are 1°, 3°,…, 101°, and the poses for Step 7 are 1°, 8°,…, 351°. Additionally, we have also investigated the possibility of training classifiers using training databases with different sizes, for example, 51 training images, 60 training images, 71 training images, and 85 training images. Features are extracted with wavelet 768 and reduced to dimensionality of 55. The results are illustrated in Figure 17.

It is noted in Figure 17 that as the incremental step of poses increases, the achieved classification accuracy grows too. More specifically, a much higher *P*_cc_ of 90.3% is achieved by employing 51 training images with incremental Step 7 in pose, in comparison with a *P*_cc_ of 42.9% achieved by employing 85 training images with incremental Step 1 in pose. The principle behind this observation is that the training datasets formed with small pose variation steps can provide less target signal information and thus, their content is not sufficient enough to cover the different appearances of the targets captured under various poses. In contrast, a much more complete training dataset can be formed when the involved images are captured with larger pose variations. The experimental results in Figure 17 show that the best classification performance 90.3% is achieved when training with 51 images captured using incremental Step 7 and 93.0% is achieved when training with 85 images captured using incremental Step 4. Moreover, it is quite straightforward to find that better classification performance is always achieved when training with relatively larger number of training images. It is worth pointing out that only a small number of images are involved in the training stage rather than several hundreds of them as used in the previous tests. This is a promising result which implies that a good classification result can be achieved even with much less number of training images, as long as they are captured with appropriate incremental step.

## 6. Conclusion

In this paper, we presented a systematic scheme for the SAR ATR task. The proposed scheme involves three main stages: preprocessing, feature extraction and processing, and classifier construction. The effectiveness of involving several preprocessing approaches (e.g., the image energy normalization and the pose rectification processes) is analysed and empirically verified. The results suggest that the involvement of these preprocessing steps is beneficial for improving the classification accuracy. Moreover, we proposed to expand the feature set to provide more information for discrimination and then eliminate the redundancy and dimensionality of the extended feature set to form a more compact and efficient feature set. Finally, the discriminative trees are learned as the base classifiers and combined to construct a strong classifier by using the Real-AdaBoost algorithm. The proposed method is evaluated with the MSTAR dataset under various operating conditions. Experimental results demonstrate that the proposed method outperforms traditional methods, for example, EMACH, SVM, NN, and IGT. The advantages of the proposed method give credit to the reduction of variations in target images, the improvement of feature efficiency, the elimination of redundancy in feature sets, and the excellent generalization capability of the combined strong classifier. Moreover, we have tested the classification performance of the classifiers trained with different combinations of target poses. Experimental results show that a classifier trained with training images covering large variations of target poses can produce good classification result even with limited number of training images.

## Figures and Tables

**Table 1:** 10 classes of targets of the MSTAR dataset under SOCs.

Training set			
Vehicle	Number of images	Serial number	Depression angle
BMP2	699	9563, 9566, C21	17°
BTR70	233	C71	17°
T72	699	132, 812, S7	17°
BTR60	256	k10yt7532	17°
2S1	299	B01	17°
BRDM2	299	E71	17°
D7	299	92v13015	17°
T62	299	A51	17°
ZILI131	299	E12	17°
ZSU234	299	D08	17°

Testing set			
Vehicle	Number of images	Serial number	Depression angle

BMP2	587	9563, 9566, C21	15°
BTR70	196	C71	15°
T72	588	132, 812, S7	15°
BTR60	196	k10yt7532	15°
2S1	274	B01	15°
BRDM2	274	E71	15°
D7	274	92v13015	15°
T62	274	A51	15°
ZILI131	274	E12	15°
ZSU234	274	D08	15°

**Table 2:** 4 classes of targets of the MSTAR dataset under EOC-1.

Training set			
Vehicle	Number of images	Serial number	Depression angle
2S1	274	B01	15°
BRDM2	274	E71	15°
ZSU234	274	D08	15°
T72	274	A64	15°

Testing set			
Vehicle	Number of images	Serial number	Depression angle

2S1	288	B01	30°
BRDM2	288	E71	30°
ZSU234	288	D08	30°
T72	288	A64	30°

**Table 3:** 5 variants of T72 with different serial numbers of the MSTAR dataset under EOC-2.

Training set			
Vehicle	Number of images	Serial number	Depression angle
BMP2	233	C21	17°
BRDM2	298	E71	17°
BTR70	233	C71	17°
T72	233	132	17°

Testing set			
Vehicle	Number of images	Serial number	Depression angles

T72	419	S7	15° and 17°
T72	572	A32	15° and 17°
T72	573	A62	15° and 17°
T72	573	A63	15° and 17°
T72	573	A64	15° and 17°

**Table 4:** MAD evaluation of the proposed method for all 10 targets at depression angle 17° (degrees).

Vehicle	BTR60	2S1	BRDM2	D7	T62	ZIL131	ZSU234	BMP2	BTR70	T72
LSM	7.05	11.05	15.48	11.93	10.47	10.79	15.42	9.39	9.33	10.22
HT	9.17	11.26	12.14	18.86	14.69	12.34	15.16	10.72	8.09	12.64
MBR	6.10	7.45	14.66	6.27	7.18	11.87	8.23	6.79	9.38	4.99
RT	7.23	9.55	10.38	19.32	13.32	9.27	18.40	9.54	7.49	13.73
Proposed method	**4.85**	**5.84**	**8.70**	**6.27**	**9.39**	**8.02**	**7.83**	**5.43**	**6.67**	**4.51**

**Table 5:** MAD evaluation of the proposed method for all 10 targets at depression angle 15° (degrees).

Vehicle	BTR60	2S1	BRDM2	D7	T62	ZIL131	ZSU234	BMP2	BTR70	T72
LSM	5.52	10.63	15.21	10.09	9.20	8.67	14.07	7.56	7.00	6.87
HT	7.00	10.16	10.96	16.72	13.49	10.13	14.32	9.02	6.87	10.38
MBR	5.11	6.64	15.32	6.40	6.93	10.23	8.81	5.12	7.01	**3.53**
RT	4.76	9.63	9.38	17.36	12.34	7.08	17.31	8.27	5.85	10.61
Proposed method	**3.81**	**5.42**	**9.04**	**6.32**	**6.10**	**5.89**	**7.67**	**4.45**	**5.13**	3.38

**Table 6:** MAD evaluation of the proposed method for all serial number variants of BMP2 and T72 at depression angle 17° (degrees).

Vehicle type	BMP2			T72		
	132	812	s7	9563	9566	c21
LSM	17.15	9.58	13.12	13.53	13.22	12.06
HT	18.87	14.81	16.23	12.96	13.04	13.76
MBR	7.97	5.50	6.40	11.90	9.96	8.71
RT	21.46	15.15	17.62	11.47	13.65	12.25
Proposed method	**7.93**	**5.42**	**5.79**	**8.47**	**7.94**	**6.96**

**Table 7:** MAD evaluation of the proposed method for all serial number variants of BMP2 and T72 at depression angle 15° (degrees).

Vehicle type	BMP2			T72		
	132	812	s7	9563	9566	c21
LSM	12.58	9.30	10.74	11.26	10.96	9.71
HT	15.98	12.36	13.32	9.44	9.63	11.58
MBR	7.75	4.85	4.53	8.57	6.93	6.57
RT	17.16	11.31	13.62	9.38	10.31	10.61
Proposed method	**7.21**	**4.60**	**4.34**	**6.96**	**5.63**	**5.71**

**Table 8:** Confusion matrix of EMACH, method proposed in [52], method proposed in [2], IGT, and the proposed method tested under SOCs (*P*_cc_ (%)).

Confusion matrix of EMACH, the method proposed in [52], and the proposed method										
Vehicle	BMP2	BTR70	T72	BTR60	2S1	BRDM2	D7	T62	ZILI131	ZSU234
BMP2	90/90/**100**	2/2/0	4/3/0	1/1/0	1/1/0	0/2/0	0/0/0	1/0/0	0/1/0	1/0/0
BTR70	2/3/0	93/90/**100**	1/3/0	0/0/0	1/0/0	1/2/0	0/0/0	2/0/0	0/0/0	0/2/0
T72	2/2/0	0/1/0	96/93/**100**	0/3/0	1/0/0	0/0/0	0/0/0	0/0/0	1/1/0	0/0/0
BTR60	0/2/0	1/2/0	0/1/2	95/92/**98**	1/0/0	0/0/0	3/3/0	0/0/0	0/0/0	0/0/0
2S1	5/5/0	6/3/0	4/2/1	2/0/0	74/81/**99**	3/3/0	1/2/0	2/3/0	1/0/0	2/1/0
BRDM2	3/6/1	6/8/0	3/2/0	0/1/0	1/0/0	84/79/**99**	2/0/0	0/3/0	0/0/0	1/1/0
D7	2/0/1	3/0/0	2/0/0	1/0/0	0/1/0	0/0/0	85/98/**99**	3/0/0	2/0/0	2/1/0
T62	1/1/0	1/0/0	1/0/0	1/1/0	4/0/1	0/0/0	0/0/0	86/91/**99**	4/4/0	2/3/0
ZILI131	2/2/0	0/1/0	1/0/0	2/0/0	0/0/0	0/0/0	0/0/0	4/0/0	88/95/**100**	3/2/0
ZSU234	1/0/0	0/1/0	4/0/1	2/3/0	0/0/0	0/0/0	0/1/0	1/0/0	0/3/0	92/92/**99**

Confusion matrix of the method proposed in [2], IGT, and the proposed method										
Vehicle	BMP2	BTR70	T72	BTR60	2S1	BRDM2	D7	T62	ZILI131	ZSU234

BMP2	92/95/**100**	2/1/0	2/1/0	0/0/0	1/1/0	2/1/0	0/0/0	0/0/0	1/1/0	0/0/0
BTR70	3/2/0	93/94/**100**	0/0/0	0/0/0	0/00/	2/2/0	0/0/0	0/0/0	0/0/0	2/2/0
T72	2/2/0	1/1/0	96/96/**100**	1/1/0	0/0/0	0/0/0	0/0/0	0/0/0	0/0/0	0/0/0
BTR60	2/1/0	0/0/0	2/1/2	93/97/**98**	0/0/0	0/0/0	3/1/0	0/0/0	0/0/0	0/0/0
2S1	3/3/0	4/4/0	1/1/1	0/0/0	87/89/**99**	2/0/0	0/0/0	2/1/0	0/0/0	1/2/0
BRDM2	5/2/1	3/1/0	2/4/0	0/0/0	0/0/0	85/90/**99**	5/2/0	0/0/0	0/0/0	0/1/0
D7	0/0/1	0/0/0	0/0/0	0/0/0	1/1/0	0/0/0	98/**99**/**99**	0/0/0	0/0/0	1/0/0
T62	1/1/0	0/0/0	0/0/0	0/0/0	0/0/1	0/0/0	0/0/0	93/95/**99**	3/3/0	3/1/0
ZILI131	2/2/0	2/2/0	0/1/0	0/0/0	0/0/0	0/0/0	0/0/0	0/0/0	94/95/**100**	2/0/0
ZSU234	1/1/0	0/0/0	0/0/1	1/1/0	0/0/0	0/0/0	0/0/0	0/0/0	2/2/0	96/96/**99**

The average *P*_cc_ of these 5 methods are sequentially 88.3%, 90.1%, 92.7%, 94.6%, and **99.3%**.

**Table 9:** Confusion matrix of EMACH, method proposed in [52], method proposed in [2], IGT, and the proposed method tested under EOC-1 (*P*_cc_ (%)).

Vehicle	2S1	BRDM2	T72	ZSU234
2S1	67/74/77/78/**99**	15/8/5/6/0	12/9/11/9/1	6/9/7/7/0
BRDM2	17/12/15/15/2	57/66/73/76/**97**	19/9/5/6/1	7/13/7/3/0
T72	7/17/11/10/0	9/6/9/9/0	66/73/75/78/**96**	18/4/5/3/4
ZSU234	10/7/4/5/1	7/5/6/5/0	2/3/2/2/1	81/85/88/88/**98**

The average *P*_cc_ of these 5 methods are sequentially 67.75%, 74.5%, 78.25%, 80.0%, and **97.5%**.

**Table 10:** Confusion matrix of EMACH, method proposed in [52], method proposed in [2], IGT, and the proposed method tested under EOC-2 (*P*_cc_ (%)).

Vehicle	BMP2	BRDM2	BTR70	T72
T72_S7	4/5/4/5/0	8/4/6/4/2	6/4/2/3/1	82/87/88/88/**97**
T72_A32	9/7/5/6/0	5/5/8/3/0	5/2/3/2/0	81/86/84/89/**99**
T72_A62	8/7/6/5/0	6/5/5/4/1	3/6/4/4/3	83/84/85/87/**97**
T72_A63	13/15/11/7/0	6/6/9/5/1	11/3/4/7/2	70/76/76/81/**97**
T72_A64	16/9/10/11/0	4/5/5/4/2	12/13/9/6/3	68/73/76/79/**94**

The average *P*_cc_ of these 5 methods are sequentially 76.8%, 81.2%, 81.8%, 84.8%, and **96.9%**.
